# Pixel-wise segmentation of cells in digitized Pap smear images

**DOI:** 10.1038/s41597-024-03566-9

**Published:** 2024-07-06

**Authors:** Balazs Harangi, Gergo Bogacsovics, Janos Toth, Ilona Kovacs, Erzsebet Dani, Andras Hajdu

**Affiliations:** 1 Department of Data Science and Visualization, Faculty of Informatics, University of Debrecen, Debrecen, Hungary; 2 Department of Pathology, Kenezy Gyula Hospital and Clinic, University of Debrecen, Debrecen, Hungary; 3 Department of Library and Information Science, Faculty of Humanities, University of Debrecen, Debrecen, Hungary

**Keywords:** Molecular imaging, Cervical cancer

## Abstract

A simple and cheap way to recognize cervical cancer is using light microscopic analysis of Pap smear images. Training artificial intelligence-based systems becomes possible in this domain, e.g., to follow the European recommendation to screen negative smears to reduce false negative cases. The first step for such a process is segmenting the cells. A large and manually segmented dataset is required for this task, which can be used to train deep learning-based solutions. We describe a corresponding dataset with accurate manual segmentations for the enclosed cells. Altogether, the APACS23 (**A**nnotated **PA**p smear images for **C**ell **S**egmentation 20**23**) dataset contains about 37 000 manually segmented cells and is separated into dedicated training and test parts, which could be used for an official benchmark of scientific investigations or a grand challenge.

## Background & Summary

Cervical cancer is one of the most common cancer types among women. In 2020, 604 000 new cases were reported worldwide, resulting in 342 000 deaths^1^. Regular screening is crucial to prevent or detect cervical cancer in its early stages. The Papanicolaou (Pap) smear has evolved into a standard screening test for cervical cancer since its efficacy was proven in 1943.

The worldwide usage of this test contributed to reductions in the incidence and mortality of cervical cancer. The specificity of cytology screening is high, but the sensitivity is still low (50–70%). The estimates for false-negative rates are from 2% to 55% according to van der Graaf and Vooijs^2^. Cytology screening demands well-trained cytologists, as the process is cumbersome, labor-intensive, and error-prone. Most commonly, errors are caused by changing the observed yield frequently and inconsistently, resulting in a subjective outcome. A re-screening quality control can reduce these mistakes, but the human resources may not be sufficient, making it high-cost. A European recommendation for cervical screening^3^ is to re-screen (second opinion) negative smears to reduce false negative cases and increase screening accuracy. This requires significant human resources and, therefore slows down the screening process, increasing the turnaround time of the finding. The bottleneck is the personnel involved in the evaluation. Therefore, re-screening is currently not or only partially feasible in some cytopathology laboratories. The smears are assessed by light microscopy with the help of cytology pre-screeners and cytopathologist specialists. Given the availability of sufficient training data, the accuracy of machine learning-based grading can often approach or even exceed that of human experts. Our dataset, which consists of the segmented and annotated cells as positive/negative, can help build a deep learning-based solution to use the visual/morphological features extracted from the input image for cell/cell group discrimination with high accuracy.

Today, the FDA-approved Hologic ThinPrep Imaging System^4^ and BD FocalPoint GS Imaging System^5^ are the most widely used semi-automated systems to assist in cervical cancer screening. Both systems use proprietary image processing techniques to diagnostically select the most relevant fields of view (FOVs). These systems do not implement a fully digital workflow: cytotechnologists can review the selected FOVs using a microscope with a motorized stage controlled by the system. More recent solutions, such as the CE-marked Datexim CytoProcessor^6^, are based on whole slide imaging (WSI) and allow a more flexible review process, e.g., by displaying the identified abnormal cells in a gallery and on the virtual slide. Several recent studies^7–11^ have investigated the application of deep learning in cervical cytology, and the first CE-marked system known to use this technique, called the Hologic Genius Digital Diagnostics System^12^, was launched in November 2021.

A large amount of high-quality annotated data with appropriate composition is required to develop reliable deep learning-based solutions for Pap test screening. However, the availability of such datasets is still limited. A few public datasets have been created for cervical cell classification like Herlev^13^, SIPaKMeD^14^, and CRIC^15^). Besides these datasets, the ISBI 2014^16^ and ISBI 2015^17^ datasets are available for cell segmentation. In 2020, the Liu *et al*. have published an overlapping cervical cell edge detection dataset (CCEDD) in^18^, which contains only 686 manually annotated images with a resolution of 2048 × 1536. The dataset Cx22 is published in^19^ and has 1320 images with a resolution of 512 × 512 and focuses on the proper instance annotation.

Segmentation of cells’ plasma and the nucleus is essential for automated screening of Pap smear tests. The related literature contains many relevant works that deal with this image segmentation problem using both conventional image processing techniques^20–22^ and deep learning-based solutions^23–25^. A reliable and independent benchmark system is required to show the usability, accuracy, and superiority of the newly proposed algorithms, as in any other field of science. The formerly mentioned ISBI 2014^16^ and ISBI 2015^17^ datasets and the related research were focusing on the appropriate segmentation of the overlapping cells. They contain only a few images: 16 cervical cytology images and 17 multi-layer cervical cell volumes. These images are published in grayscale colorspace with a resolution of 1024 × 1024 pixels and many different focal settings. Altogether, only a few hundred cells were manually annotated at the pixel-level. Although recent publications could reach very high accuracy rates, these findings have not been validated in real-world scenarios where a single specimen may contain thousands of cells. We aim to provide a realistic benchmark dataset that contains a sufficient number of annotated Pap smear images for the cervical cell segmentation problem. Such a dataset should contain enough images to train deep learning-based solutions and be sufficiently diverse and realistic for the reliable evaluation of the proposed algorithms. For this reason, we compiled an image dataset comprising 5 digitized Pap smear specimens. This dataset was segmented into 3565 segment images, each with a resolution of 2000 × 2000 pixels, encompassing approximately circa 37 000 cells in total with manually drawn annotations at the pixel-level.

## Methods

The Scientific and Research Ethics Committee of the Health Sciences Council of Hungary (see later as IRB) approved this work at the University of Debrecen, Debrecen, Hungary, through the document with protocol number OGYÉI/65989/2020. The data used for the samples are recorded anonymously and cannot be used to establish the identity of the patients. In this way, the participants were not required to provide consent for data sharing. Moreover, the IRB has waived patient consent for data sharing and delegated the right to publish data and approve this publication to the project leaders (Prof. Dr. Andras Hajdu and Dr. Ilona Kovacs). According to the agreements described above, this publication requires only the approval of at least one of these project leaders, taking into account the World Medical Association’s Helsinki Declaration and the ethical requirements for scientific publications of the University of Debrecen’s scientific application regulations.

### The clinical process

Conventional Pap smears were taken by scraping cells from the squamocolumnar junction of the cervix with the help of Cervex-Brush or Cyto-Brush. Then, the cells were spread evenly on the glass slide fixed immediately in 95% ethyl alcohol or by spray fixative. The slides were stained with Papanicolaou stain and screened by cytologists. The reporting was done using the Bethesda 2014 classification^26^.

Both negative and abnormal smears were selected during the database’s digitization, annotation, and the uploading process. For the negative smears, the pre-screening task was to select negative smears of sufficient quality (1500 cases), based on the Bethesda 2014 system criteria, with a cytopathologist performing a microscopic re-examination. Exclusion criteria: smears that were technically unsuitable were excluded. The abnormal smears were selected retrospectively. Following the abnormal result, the selection was conditioned on a positive histopathological examination: CIN2 or more severe epithelial lesion. The histological specimen had to be available at the University of Debrecen Clinical Center, Department of Pathology. They were then re-evaluated together with the previous abnormal smears. Samples were selected in case of evident positivity on histology and cytology.

### Preprocessing of digitized images

**Fig. 1:**
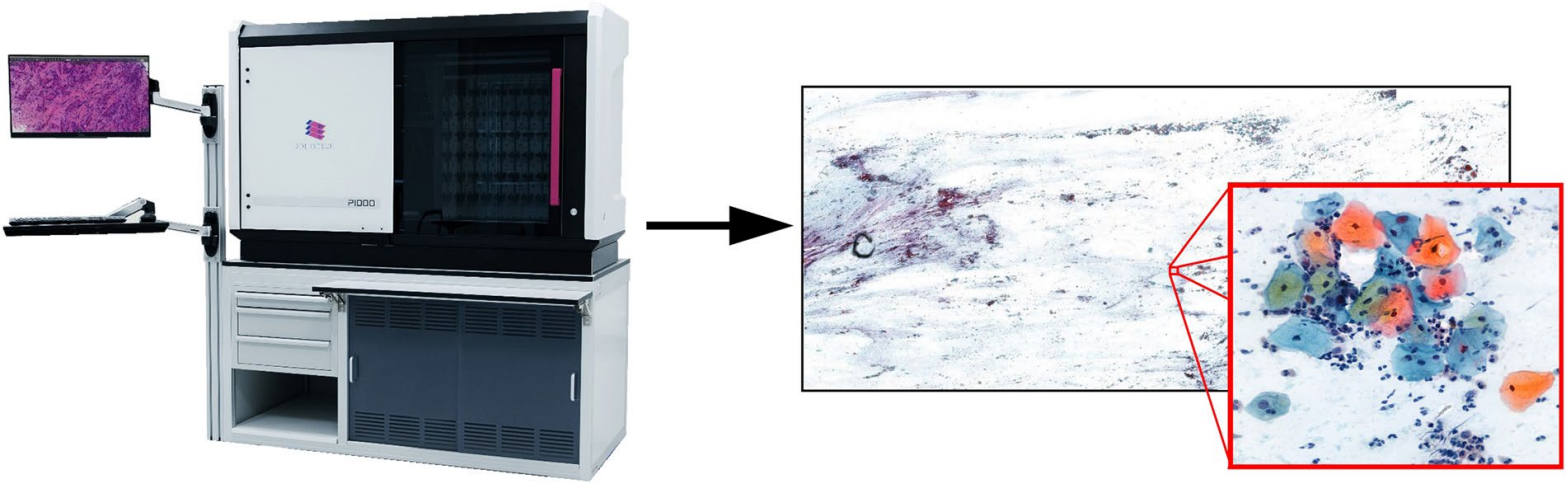
The 3D Histech scanner and the digitized Pap smear image.

Pap test slides were digitized by a laboratory analyst trained for this task by the scanner vendor using a 3DHistec Pannoramic 1000 digital slide scanner^27^ equipped with an Adimec Q-12A-180Fc brightfield camera. The laboratory analyst has properly prepared and cleaned the slides before digitizing them. When digitizing the smear, the scanner uses 3 different focal lengths (3 micrometers apart), the results of which are compressed to produce the sharpest possible image (see Fig. 1). An optical magnification of 20× was used during the scan. We have chosen this specific setting as a 20x magnification, which is commonly used in digital pathology for scanning as it balances resolution and field of view, making it suitable for various diagnostic purposes. The digitized slides were saved in MRXS format, a proprietary WSI format with multiple resolution levels for faster navigation. The dimensions of the acquired digital slides are approximately 100 000 × 200 000 pixels, and the size of a digitized slide is approximately 5 GB using JPEG compression. To extract FOVs from the digital slides, the region of interest was first identified using the intensity features of the slide at the lowest resolution level. Then, 2000 × 2000 pixels, non-overlapping FOVs were extracted from the 200× magnification level (approximately 0.25 *μ*m/pixels) of the slide as PNG files using the libvips^28^ library. This resolution and FOV size allow the examination of the patterns at the cellular level, the context of a cell, and the arrangement of cells. The scanning analyst recorded and anonymized the non-personal clinical data (age, Bethesda classification scoring category, smear serial number) required for subsequent processing.

To build the dataset, we used specimens from 5 patients previously treated at our local clinic. All the smear tests contain completely healthy cells besides cells showing signs of lesions. At the beginning of the database design, it was determined which 3 images from the 5 will be included in the training set and which 2 will be in the test set to ensure the complete independence of the two parts. As a next step, all 5 images were split into 2000 × 2000 pixels sub-images, so a total of 3565 image slices were extracted. Considering the training and test parts, 2227 image slices were placed into the training set and 1338 into the test one. Manual drawing was performed on these extracted images, and all the contained cells were annotated.

### Manual annotation of cells

The manual annotation of the cytological smears was carried out by a team of three annotators coordinated by a team leader. The annotators all had excellent digital skills and different biological education levels. Prior to the actual annotation task, there was a comprehensive training program in IT and cytology, consisting of different phases built on each other.

In the first stage (three sessions), annotators were introduced to computer image editing using the free licensed graphics software Paint.net 4.3.4, with the default PDN format, which is a compressed representation of the image layers and other information used by the program. This graphical editor was chosen because, despite its limited features, it was easy to use in all aspects and allowed the essential tasks for the processing of cytological images. One such task was the layering function, which allows one to work with different layers of images.

In the second phase of the training (five sessions), the laboratory workflow for cytological smears was described in a compact form. The annotators had to acquire minimal knowledge to carry out this work. Three sessions were devoted to clarifying the basics of cellular biology and histology, explaining the cellular composition of smears with illustrations and concrete examples in each case. They had to identify the cells annotated in the cell smear as accurately as possible, and they were required to distinguish between cell components and possible impurities, such as red blood cells, salivary cells, mitochondria, bacteria, etc. We paid attention to making the annotators familiar with the Papanicolaou staining technique since the quality of staining, apart from changing the cell morphology and the nucleus-plasma ratio, can play a significant role in misinterpreting the cell image. We concluded this phase with control exercises.

The third phase could be considered as a pilot, during which each annotator had to process 50–50 images in the graphics software. The team leader reviewed the annotations completed within the time limit and returned them to the annotators with the possible errors marked. The most common errors were:

- interpreting other components as squamous cells;
- not recognizing the cell due to a blurred cell wall and therefore not labeling it;
- not labeling all the cells that were superimposed when processing in multiple layers;
- regularly interpreting red blood cells as squamous cells.

After the two-month preparatory phase, the team leader developed a specific schedule for the annotation process as follows:

- 4 months time frame;
- 3565 images;
- 453 re-annotations after verification (typically due to error rates in multi-layer processing);
- 246 unassessable images due to insufficient staining;
- approximately half of the processed images were produced in multi-layer processing.

We found that the repetitive and monotonous nature of the annotation work, coupled with the potential for extended work sessions lasting several hours, significantly increased the error rate. This was already apparent in the first month, so the group leader asked the annotators to submit their work weekly, asking them to spread the annotation work evenly, preferably over each day, up to one hour. The first hour of concentrated attention meant significantly fewer incorrect or missed cell annotations.

**Fig. 2:**
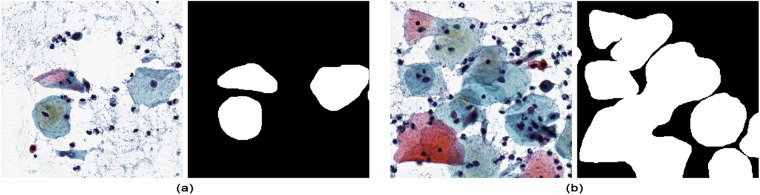
Manually drawn segmentation for (**a**) single cells and (**b**) cell conglomerates.

This properly controlled annotation process resulted in a manual annotation of 3565 images at a resolution of 2000 × 2000 pixels. For each image slice, the annotation is published as a binary image where the area of the cell’s cytoplasm is marked as white (the nuclei are not shown separately, as it can be seen in Fig. 2a), and black pixels represent every other part. The cluster of cells, where they overlap on the digitized smear from the point of scanning view, are drawn with outlines representing their outer boundaries. Consequently, these cell conglomerates are composed of a single object, and the individual cells within these groups are not marked separately (see Fig. 2b).

### Automatic segmentation

**Fig. 3:**
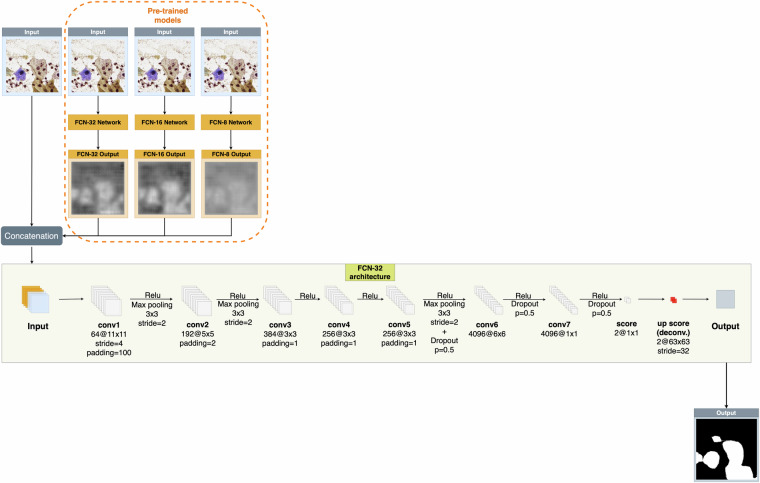
The proposed framework from our original paper^10^. Our fusion-based system receives the heatmaps of the individual FCN algorithms and the original RGB image as input (left) to generate the segmented output image (right).

After manually annotating the images, we trained several deep convolutional neural networks on an initial version of the dataset, which we discussed in our former work^10^. We aimed to develop reliable neural network-based solutions that could automatically segment the cells from the background with high accuracy. To accomplish this, we proposed a fully automatic region-based neural network ensemble architecture in our original paper^10^ that greatly outperformed both traditional aggregation methods, such as majority voting or statistical combination and other state-of-the-art neural network architectures as well. Our method used a novel Fully Convolutional Neural Network (FCN)^29^ as its backbone, which received both the outputs of some pre-trained models and the original input image. Namely, we pre-trained the FCN-8, FCN-16, and FCN-32 models on the same dataset, and our fusion model further processed the heatmaps generated by each model in conjunction with the original RGB input image. Our reasoning behind this method was that we had observed several cases where different FCN networks (FCN-8, FCN-16, and FCN-32) gave very different and distinct outputs and combining them led to better results. In our work^10^, we have shown that using this approach resulted in the best, most accurate segmentation results for our dataset. An overview of our fusion-based approach can be seen in Fig. 3.

For the training of our solution, we trained several variants of our proposed architecture on the data and then measured their performances on the test set. To evaluate the trained networks, we used the following metrics: true positive (TP), false positive (FP), true negative (TN), and false negative (FN). TP denotes the number of pixels representing the location of cell clusters in the output and the original mask. TN is similar to TP but refers to the number of pixels that belong to the background. FP and FN are the number of pixels incorrectly identified in the output. Based on these values, the following metrics were computed for each algorithm: Accuracy, Intersection over Union (IoU)^30^, and Dice score (DSC)^30^. These metrics were selected from a more extensive list of potential metrics because a thorough analysis of the results indicated that they most accurately represented the performance of the algorithms. This meant that if a given segmentation result was deemed visually suitable, these metrics were also very high and vice-versa. We validated our findings using the expanded dataset featured in this paper and we used the images of size 2000 × 2000 pixels during the process. We evaluated the performance of our architecture from our previous work^10^ using the test set of this dataset. During this final evaluation process, it was found that our combined framework performed reliably in all cases, achieving 97.76% Accuracy, 57.93% IoU, and 73.36% DSC. The framework outperformed all of the individual FCN algorithms, namely the FCN-32, FCN-16, and FCN-8 networks, which achieved 97.40%, 97.39%, and 97.51% Accuracy, 56.30%, 49.34%, and 52.41% IoU, and 72.04%, 66.08%, and 68.77% DSC, respectively.

Despite the increased complexity and number of computations, we measured that training the ensemble took only 48% longer than that of the original FCN-32 algorithm while still outperforming the latter considerably: by 1.63% and 1.32% in terms of IoU and DSC. In the case of the inference part, the differences were even more minor: the running times of the individual members and their ensemble were almost the same. The exact times to provide the outputs of the FCN-32, FCN-16, FCN-8, and our combined network for a given batch of 4 images were 1.2012, 1.4464, 1.4288, and 1.4688 seconds, respectively. These results suggest that the presented method does not suffer from any time-related drawbacks or constraints that would compromise its usability in clinical practice.

**Fig. 4:**
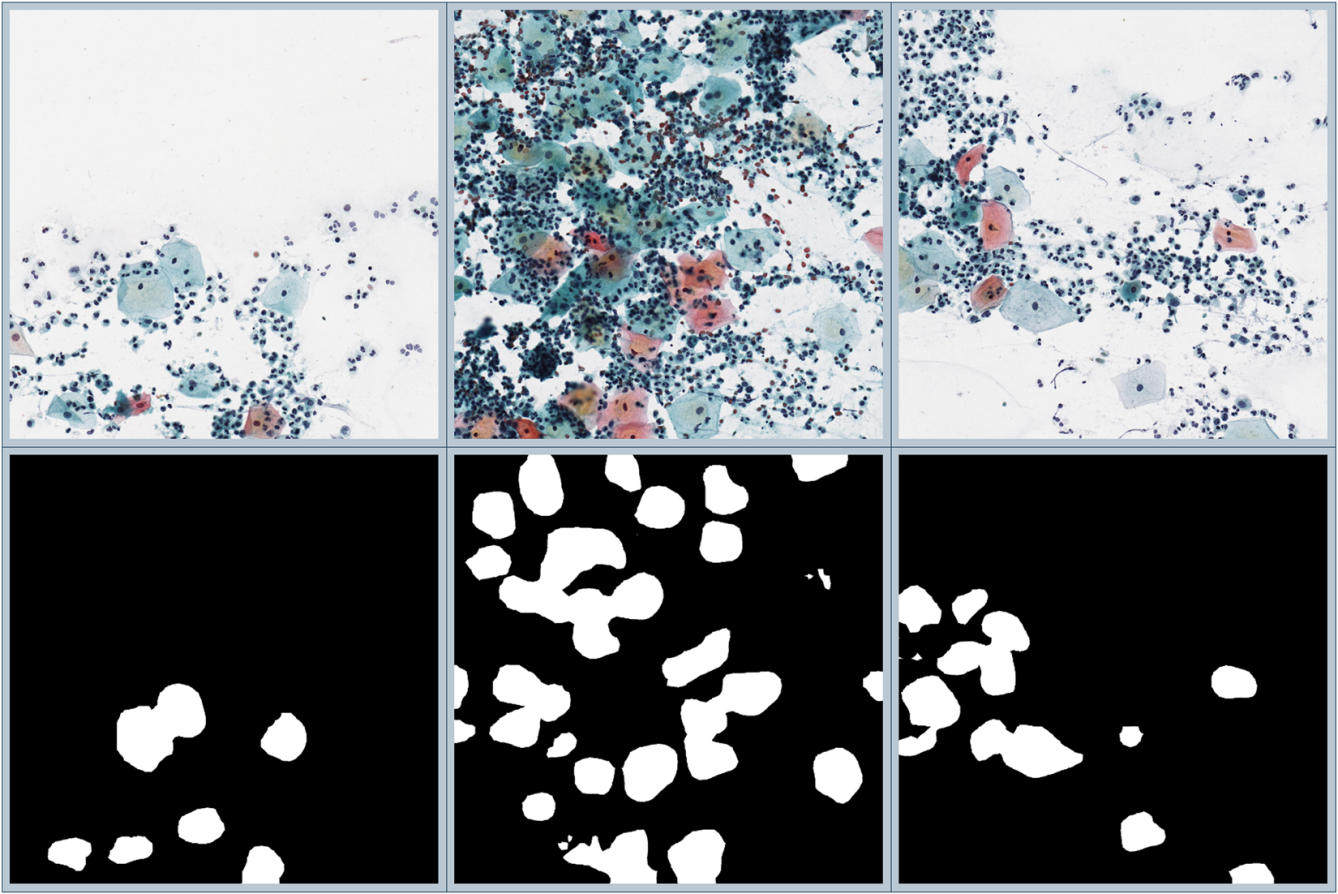
Some sample input images (top row) and the outputs of our proposed framework^10^ (bottom row).

For an overview of the segmentation results of the proposed automatic method on our proposed dataset, see Fig. 4.

## Data Records

All annotated images are available as the APACS23 dataset^31^ under the Creative Commons Attribution 4.0 International (CC BY 4.0) license deposited in an Open Science Framework (OSF) repository.

We have split the images into a training and a test set to ensure appropriate tools for training and evaluating the algorithms for the automated segmentation task. This clear splitting aims to be used in any further development related to the field for an official evaluation as a benchmark. Moreover, we plan to organize an international challenge to make a reliable evaluation and performance measurement for the participants.

As indicated in the previous sections, the image slices from 3 smears were included in the training data set (2227 images), and image slices from 2 smears were included in the test data set (1338 images). Considering the number of cells, the training dataset contains circa 30 000 cells, while the test dataset contains nearly 7 000 cells. Therefore, the full downloadable dataset includes a training and a test part as seen in Table 1.

**Table 1:** Details of the published dataset.

Training part		Test part	
3 different Pap smears		2 different Pap smears	
30 000 cells		7 000 cells	
Input	Ground Truth	Input	Ground Truth
2227 RGB images	2227 binary masks	1338 RGB images	1 338 binary masks

The input images are saved as three-channel RGB digital color images with a resolution of 2000 × 2000 pixels in JPEG format. The manual annotation for any input image is saved with the same name as a single-channel binary image in PNG format. As shown in Table 1, the total downloadable dataset contains 7 130 files organized into 4 folders with a total size of 1.6 GB.

## Technical Validation

Following the automated annotation process, the team leader manually re-annotated the images in the test dataset, taking into account the algorithm’s output. This step serves as a form of technical validation or can be seen as a hybrid annotation approach involving both machine and human input. In cases, where the algorithm fails to find a manually annotated cell or incorrectly detects images that are not classified as cells, the following problems may systematically be the cause:

- poor quality of staining/differentiation: faint or over-staining, altered cell morphology or nucleus-to-plasma ratio;
- visual field clogged with bacteria or contamination;
- staining problems of cellular components and tissues;
- thick, bloody, fragmented visual field.

Another significant issue arose when cells were located at the edges of the visual fields, which were only partially visible (usually consisting of cytoplasm/cell wall fragments without nuclei), were decided to be excluded from manual annotation. However, the algorithm detects these cells with a high degree of accuracy.

## Usage Notes

Given the clinical nature of the data, the dataset is published under the Creative Commons Attribution 4.0 International (CC BY 4.0) license to facilitate the implementation and integration of reliable computer-aided diagnosis (CAD) systems in the clinical workflow for early cancer detection. Any special inquiries or requests regarding the re-distribution, modification, transformation, or use of the data should be directed and communicated to the authors.

Due to the nature of our model, that is, using the outputs of other models in addition to the input image, the total wall time required to generate the segmentation masks for a given image has also significantly increased. This is also true for the training process, where using any number of pre-trained models means that we first need to pass the input image through these models before passing them to the ensemble model. In an attempt to alleviate this problem, the proposed framework reads each input image only once to reduce the number of I/O operations, stores the image in memory, and passes it to each individual FCN algorithm as well as to the ensemble model. In the future, we also plan to experiment with additional mechanisms and techniques to speed up this process.

## Data Availability

We have made our framework available on GitHub via the following link: https://github.com/gergobogacsovics/APACS23; it is capable of the automatic segmentation of Pap smear images using the combined architecture from our original paper^10^. We made all the corresponding codes and helper scripts, responsible for data reading, pre-processing, training, and testing, freely available under the GNU General Public License v3.0 in an attempt to facilitate any related research as well as the spread of reliable, modern AI-assisted clinical image-processing systems, and to also ensure the open nature of future research.
